# Functional Nanomaterials for Sensing Devices: Synthesis, Characterization and Applications

**DOI:** 10.3390/nano14201652

**Published:** 2024-10-15

**Authors:** Barbara Vercelli

**Affiliations:** Istituto di Chimica della Materia Condensata e di Tecnologie per l’Energia, CNR-ICMATE, Via Cozzi, 53, 20125 Milano, Italy; barbara.vercelli@cnr.it

## 1. Introduction

The growing field of nanotechnology impacts many research areas, such as engineering, electronics, energy, environment, biology, and medicine [1,2].

“Nanotechnology” is the discipline that deals with nanomaterials: the materials whose dimensions are smaller than 100 nm [3,4]. When materials are in the nanoscale dimension, their physicochemical properties (like optical, electrical, chemical, strength, potential, etc.) are affected by the “nano-size” compared to the bulk macroscale counterparts [5]. The high surface area to volume ratio and dominating quantum confinement explain the observed change in properties [4,5]. This advancement in material science has enabled the miniaturization of bulky devices and appliances, be it a transistor or an imaging system [6].

Sensor technology was also affected in terms of improvement of measurement accuracy and/or device sensitivity [7]. Sensors have benefitted hugely from nanomaterials because of the reduced amounts of sample required for the sensing, which reduces the problem of not having enough analyte to detect [8]. In recent years, nanotechnology and nanomaterials have allowed previously unimaginable advances in the development of sensor systems [9,10,11,12]. Depending on the sensing method and the analyte, they enabled a marked reduction in the detection limit.

Future challenges for the design of efficient sensors for large-scale industrial applications mainly regard the improvement of specificity, reproducibility, and the ability to detect trace levels. Recent developments in nanotechnology, aimed at realizing a new class of functional nanomaterials based on bottom-up assembly of nanoarchitectures [13], are expected to give a considerable impulse to overcoming those challenges.

## 2. An Overview of the Published Articles

La Ferla et al. (Contribution 1) discussed the limits and the problems encountered in employing hydrothermal synthetic strategies for the sustainable preparation of red-emitting carbon quantum dots (r-CDs) for advanced medical applications. They suggested that knowledge of those issues may stimulate further studies aimed at synthesizing long-wavelength CDs, controlled according to their purpose, for precision medicine applications.

Chen et al. (Contribution 2) realized for the first time an all-fiber temperature sensor based on MXene V2C. They employed different techniques to characterize the obtained material, which improved the device sensing efficiency (0.32 dB/°C).

Cho et al. (Contribution 3) realized a Sandwich Structure Piezoresistive Woven Nanofabric (SSPWN) based on a stretchable metal–organic nanocomposite comprising reduced graphene oxide (rGO) and in situ generated silver nanoparticles (AgNPs). rGO is blended with elastic electrospun polystyrene-butadiene-polystyrene (SBS) fibers to obtain a tactile-sensitive wearable sensor. The sensor exhibits stability in 55,000 cycles and a response time of less than milliseconds.

El Habra et al. (Contribution 4) employed MOCVD to grow TiO_2_ thin films on modified stainless-steel mesh for sensing applications. They used different techniques to analyze both the substrate and the deposited TiO_2_. The detection properties of TiO_2_ thin films as COD sensors were discussed through photocatalytic degradation tests on functional model pollutants based on ISO 10678:2010.

Vidal et al. (Contribution 5) described an easy and low-cost way to fabricate monometallic Au nano-islands for plasmonic-enhanced spectroscopy. The method is based on the direct thermal evaporation of Au on glass substrates to form nano-islands, which are subsequently covered by a thin layer of silicon dioxide. The authors reported an exhaustive characterization of the obtained photo-plasmonic substrates and showed their applicability in confocal fluorescence and Raman microscopies.

Quiroz-Arturo et al. (Contribution 6) reported polymer-modified glassy carbon electrodes for the detection of metronidazole. They employed characterization techniques supported by theoretical calculations to unveil the reasons behind the high efficiency of electrochemical detection of metronidazole.

## 3. Conclusions

In conclusion, the contributions to this Special Issue give an overview of the state-of-the-art synthesis and development of sensor nanomaterials. The discussed arguments range from issues related to the reliable and sustainable synthesis of red-emitting dots with reproducible and robust properties for advanced medicine applications to stretchable nanocomposites for tactile-sensitive wearable sensors. The focus on developing nanomaterials for environmental pollution sensors is also covered in this issue. The efficient synthesis of temperature-sensitive nano-systems, metal oxide thin films for COD sensors, and polymer-modified electrodes for water treatment may significantly contribute to environmental applications. Furthermore, the fabrication of monometallic nano-islands for advanced plasmonic applications is also discussed. All the works are valuable contributions to this Special Issue. They are expected to stimulate further work and discussion in the field, promoting a step forward in the design and synthesis of efficient and sustainable nanomaterials for sensor applications.
